# Sulforaphane acutely activates multiple starvation response pathways

**DOI:** 10.3389/fnut.2024.1485466

**Published:** 2025-01-06

**Authors:** Kendra S. Plafker, Constantin Georgescu, Nathan Pezant, Atul Pranay, Scott M. Plafker

**Affiliations:** ^1^Aging and Metabolism Research Program, Oklahoma City, OK, United States; ^2^Genes and Human Disease Research Program, Oklahoma City, OK, United States; ^3^Center for Biomedical Data Sciences, Oklahoma Medical Research Foundation, Oklahoma City, OK, United States

**Keywords:** sulforaphane, starvation, autophagy, mTOR, Txnip, Sestrin 2

## Abstract

Sulforaphane (SFN) is an isothiocyanate derived from cruciferous vegetables that has demonstrated anti-cancer, anti-microbial and anti-oxidant properties. SFN ameliorates various disease models in rodents (e.g., cancer, diabetes, seizures) that are likewise mitigated by dietary restrictions leading us to test the hypothesis that this compound elicits cellular responses consistent with being a fasting/caloric restriction mimetic. Using immortalized human retinal pigment epithelial cells, we report that SFN impacted multiple nutrient-sensing pathways consistent with a fasted state. SFN treatment (i) increased mitochondrial mass and resistance to oxidative stress, (ii) acutely suppressed markers of mTORC1/2 activity via inhibition of insulin signaling, (iii) upregulated autophagy and further amplified autophagic flux induced by rapamycin or nutrient deprivation while concomitantly promoting lysosomal biogenesis, and (iv) acutely decreased glucose uptake and lactate secretion followed by an adaptive rebound that coincided with suppressed protein levels of thioredoxin-interacting protein (TXNIP) due to early transcriptional down-regulation. This early suppression of TXNIP mRNA expression could be overcome with exogenous glucosamine consistent with SFN inhibiting glutamine F6P amidotransferase, the rate limiting enzyme of the hexosamine biosynthetic pathway. SFN also altered levels of multiple glycolytic and tricarboxylic acid (TCA) cycle intermediates while reducing the inhibitory phosphorylation on pyruvate dehydrogenase, indicative of an adaptive cellular starvation response directing pyruvate into acetyl coenzyme A for uptake by the TCA cycle. RNA-seq of cells treated for 4 h with SFN confirmed the activation of signature starvation-responsive transcriptional programs. Collectively, these data support that the fasting-mimetic properties of SFN could underlie both the therapeutic efficacy and potential toxicity of this phytochemical.

## Introduction

Sulforaphane (SFN) is an organosulfur compound derived from vegetables belonging to the *Brassicaceae* family (e.g., kale, broccoli, Brussels sprouts). Studies over the past three decades have established the therapeutic potential of SFN, determined a clinical history and safety profile for the compound, and demonstrated that SFN can be readily combined with other interventions (1, 2).

The pharmacological efficacy of SFN has largely been characterized in the context of stabilizing and activating NF-E2-related factor 2 (Nrf2), a master anti-stress and anti-oxidant transcription factor. SFN-mediated activation of Nrf2 induces the expression of a battery of genes encoding proteins that maintain and restore redox and proteome homeostasis (reviewed in (3, 4)). Nrf2 activation additionally shunts glucose into anti-oxidant pathways by inducing the expression of the rate limiting enzymes for the pentose phosphate pathway, the glucuronidation pathway, and nucleotide biosynthesis. This redirection of glucose into anabolic pathways generates molecules for neutralizing oxidative stress and eliminating xenobiotics, for replacing damaged proteins and DNA, and for restoring homeostasis (4).

Nrf2 activation by SFN has been studied intensively in the context of mitochondrial impacts including a body of work related to the capacity of the phytochemical to counter neurodegeneration [e.g., (5)]. SFN can preserve mitochondrial membrane potential by increasing the resistance of mitochondria to the deleterious consequences of redox imbalances stemming from unchecked reactive oxygen species (ROS) [e.g., (6)] and dopamine toxicity [e.g., (7)]. Moreover, SFN preserved mitochondrial function, induced mitochondrial biogenesis [e.g., (8–11)] and mitigated pathologies in rodent models of brain injury, spinal cord injury, stroke, Alzheimer’s disease, Parkinson’s disease, Huntington’s disease, depression, and multiple sclerosis [reviewed in (12)].

In the work presented here, we investigated the hypothesis that SFN is a fasting mimetic at the cellular level. Our rationale was twofold. First, evidence from both animal and human studies have shown that caloric restriction, fasting regimens, timed feeding, and low carbohydrate diets can extend lifespan, slow age-related declines in muscle loss and cognitive function, and prevent or mitigate a variety of chronic diseases (13). The *in vivo* efficacy of these dietary strategies includes improvements in fasting blood glucose, fasting insulin, triglycerides, C-reactive protein (CRP), fibroblast growth factor 21 (FGF21), circulating branched chain amino acids, adiposity, and blood pressure, concomitant with increased stress resistance, altered redox metabolism, reduced inflammation, and reduced numbers of auto-reactive immune cells (13). At the organismal and cellular level, these nutritional approaches converge to modulate proliferation cascades, proteome homeostasis, stress responses, and central metabolic nodes (13). Importantly, SFN elicits many of these same responses *in vivo* [e.g., (11, 14–19)]. Second, we previously demonstrated that SFN induces a protective mitochondrial hyperfusion phenotype (20) similar to what was observed in cells challenged with complete nutrient deprivation (21). Together, these *in vitro* and *in vivo* observations led to us to test if SFN phenocopied other aspects of cellular starvation involving mTOR signaling, autophagy, lysosomal and mitochondrial biogenesis, and signature transcriptional responses to starvation.

To extend our previous work with SFN and Nrf2 in cultured cells (20, 22–24) the studies of this report were done in telomerase-immortalized human retinal pigment epithelial cells (RPE-1 cells), which we have shown rapidly and robustly respond to SFN treatment in both Nrf2-dependent (20) and Nrf2-independent ways (24). The use of RPE-1 cells additionally enabled us to take advantage of several stably-transfected RPE-1 reporter cell lines we previously generated and characterized for tracking dynamic changes in mitochondrial redox status, mitophagy, and autophagy (20, 25). In the current work, we characterized the impacts of SFN on nutrient-sensing pathways using 25 *μ*M SFN, a concentration within the range detected in the serum of numerous human and animal studies studying the compound [e.g., (26)]. Our assays use early time points (e.g., 1, 4, and 8 h) to interrogate acute direct effects of the phytochemical on different cellular pathways and later time points (i.e., 16 and 24 h) to detect cellular adaptations to these acute impacts. The data presented here test whether SFN induces a fasted cellular state, in the presence of sufficient nutrients, characterized by reduced pro-proliferative signaling, increased autophagic flux, increased lysosomal biogenesis, as well as altered glucose and pyruvate metabolism.

## Materials and methods

### Chemical, reagents, antibodies

A comprehensive list of the chemicals, reagents, and antibodies used, along with the companies these were acquired from, the catalog numbers, and the concentrations/dilutions used are provided in Supplementary Table S1.

### Cell culture

Telomerase-immortalized, human retinal pigment epithelium cells (RPE-1, ATCC CRL-4000) were cultured as described (20). Briefly, cells (passage ~8–24) were plated to ~70% confluency in complete DMEM (1 g/L glucose supplemented with 1X non-essential amino acids and 10% (v/v) heat-inactivated fetal calf serum (FCS)) 1 day before experimental treatments. RPE-1 cells stably expressing GFP-LC3, mtKeima and mito-roGFP have been described previously (25). Starvation media was as follows: no glucose (Corning cat#17-207-CV no glucose DMEM supplemented with pyruvate, glutamine, non-essential amino acids, and 10% (v/v) dialyzed-heat-inactivated fetal calf serum (dFCS)), no serum (Corning, 17-207-CV supplemented with 1 g/L glucose, pyruvate, glutamine and non-essential amino acids), no amino acids or Ringer’s (Alfa Aesar Kreb’s-Ringer bicarbonate-buffered solution cat# J67591 with or without 10% (v/v) dFCS). siRNA transfection was performed as previously described (24). 25 *μ*M SFN treatment was used exclusively throughout this manuscript for the indicated incubation times.

### Flow cytometry

GFP-LC3, mito-RoGFP, mtKeima, LTR, and MTG/TMRE flow cytometry were done as described (25), and FlowJo 10.3 software was used for gating analysis and quantification for all flow cytometry studies.

Mitochondrial membrane potential was determined in RPE-1 cells treated with vehicle (DMSO), SFN, or different nutrient deprivations for 4 or 24 h and co-labeled during the final 30 min of treatment with 5 nM tetramethylrhodamine ethyl ester (TMRE) and 200 nM MitoTracker Green™ (MTG). Cells were trypsinized, resuspended in complete media lacking Phenol Red, filtered through 100 μm mesh and subjected to flow cytometry. MTG was visualized with a 488 nm laser and a 510/21 nm filter. TMRE was visualized with the 561 nm laser and a 582/15 nm filter. Membrane potential changes were calculated using the ratio between the TMRE (red) and MTG (green) signals. Mitochondrial mass of RPE-1 cells treated for 24 h with either vehicle (DMSO), SFN, or different nutrient deprivations was measured by incubating cells in 200 nM MTG for the final 30 min of the 24 h treatments. Cells were processed for flow cytometry and mitochondrial mass was calculated by median fluorescence intensity (MFI) and graphed as fraction of control.

To assess mitochondrial redox stress, RPE-1 cells stably expressing mito-roGFP were treated for 4 h with SFN, FCCP, or different nutrient deprivations and then processed for flow cytometry using the 405 nm laser with 525/50 nm filter to visualize oxidized mito-GFP and the 488 nm laser with 530/30 nm filter to visualize reduced mito-GFP.

To quantify mitophagy, RPE-1 cells stably expressing mitochondria-targeted (mt)-mKeima and YFP-parkin were treated for 4 h at 37°C with vehicle, SFN, different nutrient deprivations or FCCP. Cells were then trypsinized, resuspended in 10% fetal calf serum (FCS) and 0.5 mM EDTA in PBS, passed through 100 μm mesh, and subjected to flow cytometry. YFP-Parkin positive cells were detected with 488 nm excitation and a 530/30 nm emission filter. Mt-mKeima was detected with a 488 nm excitation laser and a 695/40 nm emission filter for mitochondrial localization, and a 561 nm laser with 670/30 nm filter for lysosomal localization. YFP-Parkin-positive cells were plotted in FlowJo as acidic against neutral and two gates defined the cells exhibiting predominately lysosomal or mitochondrial mt-mKeima localization.

For autophagy assays, RPE-1 cells stably expressing GFP-LC3 were treated with SFN, the indicated pharmacological treatments, or starved of various nutrients for 24 h. Cells were then trypsinized, resuspended in 0.05% (v/v) Saponin/PBS to leak out soluble GFP-LCS not associated with autophagolysosomes, pelleted at 500 × g for 5 min, resuspended in PBS, filtered and subjected to flow cytometry.

Lysosomal mass was quantified by LysoTracker Red (LTR) uptake in RPE-1 cells treated with vehicle, SFN, Rapa, or different nutrient deprivations for 16 h. Cells were incubated with 50 nM LTR for the final 30 min of the 16 h. Cells were resuspended in PBS + 2% FCS and subjected to flow cytometry using the 561 nm laser and 582/15 nm filter.

### Western blotting

Western blotting was performed as previously described (22, 23, 25) with the antibodies listed in Supplementary Table S1. Samples were solubilized in 2-times concentrated Laemmli buffer (100 mM Tris,pH 6.7; 2% (w/v) SDS; 25% (v/v) glycerol; 0.001% (w/v) Pyrinin Y; 0.008% (w/v) Bromophenol Blue; 285 mM b-mercaptoethanol) and heated in a boiling water bath for 5 min prior to being loaded onto gels for reducing, denaturing SDS-PAGE. All depicted Western blots are representative of at least 3 independent experiments and the migration of molecular weight markers (kDa) are indicated to left side of blots.

### qPCR

RNA was isolated using the *Zymo Direct-zol microRNA Isolation Kit and cDNA* was produced using the Quanta qScript cDNA Supermix, both per manufacturers’ instructions. qPCR was performed with PowerUp SYBR Green (Applied Biosystems, Inc.) using the primers listed in Supplementary Table S2.

### Lysosome labeling

Lysosomes were visualized in live cells by incubating in 50 nM LysoTracker Red for 30 min.

Unincorporated dye was removed by replacing the media and images were captured on a Nikon TE2000 inverted microscope as described (25).

### Immunofluorescence

RPE-1 cells were plated in 12-well dishes containing sterile glass coverslips. After the indicated treatments, cells were fixed in 4% (w/v) paraformaldehyde/PBS for 20 min, permeabilized in 0.2% (v/v) Triton X-100/PBS for 5 min, blocked in 3% (w/v) bovine serum albumin/PBS and probed overnight with anti-pPDH1 diluted 1:250. The following day, cells were washed in PBS and incubated in Alexa_488_-conjugated donkey anti-rabbit IgG and Hoechst 33342, mounted and sealed on microscope slides, and imaged as described (22, 23, 25).

### Glucose uptake and lactate secretion assays

Following treatment with DMSO, 25 *μ*M SFN or 5 mM 2-deoxy-d-glucose (2-DG) for the indicated times, RPE-1 cells were washed with warm media lacking both serum and glucose, incubated with Glucose Uptake Probe-Green (Dojindo,Inc. catalog# UP02) diluted 1:500 in glucose-and serum-free media for 15 min, washed with ice cold 1X WI buffer (Dojindo, Inc.), scraped into 1X WI buffer, filtered through 70 *μ*m mesh, and subjected to flow cytometry on a BD FacsCelesta cell sorter equipped with a 488 nm laser and processed with Flowjo software.

For assessing lactate secretion per 10,000 cells, RPE-1 cells were plated in 24 well plates and incubated with 25 *μ*M SFN or DMSO vehicle for 8 h after which media was removed for analysis (initial 8 h measurement) and replaced with fresh media containing 25 *μ*M SFN or DMSO vehicle for an additional 16 h (for a total of 24 h treatment). Media was again removed for analyses (final 16 h measurement). Both the initial 8 h and final 16 h media collections were subjected to centrifugation at 4°C (500xg for 5 min) to remove cellular debris. Clarified supernatants were then snap frozen in liquid nitrogen. To determine cell number, remaining adherent cells were fixed and stained with 0.5% (w/v) crystal violet in 25% (v/v) methanol for 20 min, and then washed, air-dried, dissolved in methanol and the OD_570_ was read and compared against a standard curve made using known numbers of cells. The lactate concentration in the previously snap frozen media was assayed using the L-Lactate Assay Kit I (Eton Biosciences, Inc.) and normalized to cell number.

### Metabolomics

RPE-1 cells (1.5×10^6^ for LC/MS and 0.5×10^6^ for GC/MS, 5 replicates/treatment/analysis) in complete DMEM were treated with vehicle or 25 *μ*M SFN for 4 h. Cells were then washed with ice cold PBS before being snap frozen in liquid nitrogen. LC–MS analysis was performed as previously published (27) on an Agilent 6,546 LC/Q-TOF coupled to an Agilent 1,290 Infinity II LC. Chromatographic separation was performed on an Agilent InfinityLab Poroshell 120 HILIC-Z, 2.1 × 150 mm, 2.7 μm column, coupled with a UHPLC Guard, HILIC-Z, 2.1 mm × 5 mm, 2.7 μm, at 15°C, with a total run time of 29 min. GC/MS was performed as previously described (28).

### Co-immunoprecipitation assay

RPE-1 cells were treated with vehicle or 25 *μ*M SFN for 4 h before being lysed on ice in TPER (Thermo Scientific, Inc. catalog# 78510) supplemented with a proteinase inhibitor cocktail containing AEBSF hydrochloride, aprotinin, bestatin, E-64, leupeptin hemisulfate, and pepstatin A (EZBlock™, Biovision, Inc). Insoluble cellular debris was removed by centrifugation at 4°C (16,000 x g for 15 min) and soluble, extracted MondoA/MLX was captured by overnight incubation at 4°C with an anti-MLX antibody. Immunoprecipitates were washed, solubilized, and resolved by denaturing, reducing SDS-PAGE. MondoA was visualized with anti-MondoA antibody.

### Chromatin immunoprecipitation assay

CHIP was performed as described in (29). 1×10^7^ RPE-1 cells were treated with vehicle or 25 *μ*M SFN for 4 h, trypsinized, washed and fixed in 1% (w/v) formaldehyde in PBS at room temp for 5 min before the reaction was stopped by the addition of 125 mM glycine. Cells were washed and DNA was sheared using a Covaris E220. Chromatin DNA (chDNA) was mixed with anti-MondoA (MLXIP) antibody and Protein G magnetic beads overnight at 4°C. Bound chDNA was eluted, cleaned up with Zymo CHIP DNA Clean and Concentrator and used for CHIP qPCR with the following primers: TXNIP-CHIP7: 5’-CTC GCG TGG CTC TTC TG-3′ and TXNIP-CHIP8: 5’-GCA GGA GGC GGA AAC GT-3′. ΔCt was calculated by normalizing to input chDNA.

### RNA-seq

RPE-1 cells (50,000 cells/replicate, 5 replicates/treatment) in complete DMEM were treated with vehicle or 25 *μ*M SFN for 4 h. RNA was isolated as described above and sequenced in the OMRF Clinical Genomics Center. The Lexogen QuantSeq sequencing data was aligned using a pipeline established by Bluebee, the Lexogen associated data analysis tool. Ensemble gene IDs were annotated using the biomart library in R for the GRCh38 (human) build. Read-count normalization and differentially expressed analyses were performed using the edgeR package from Bioconductor. Expression values quantile normalized with the voomWithDreamWeights function were analyzed for differential expression, accounting for repeated measure dependencies, using the standard functions of the limma package. Moderate t-test *p*-values were adjusted for multiple testing using the false discovery rate (FDR) method and FDR (q.value) <0.05 was used to filter significant differences. For a comparative look at fasting effect, raw fastq files and count data on HeLa cells under nutrient starvation protocol were downloaded from GEO repository, (accession code GSE211066), processed and analyzed alongside our sequencing data. Differentially expressed genes (DEGs) were defined by having an adjusted *p*-value (*q*- value) < 0.05 and a log2FC < −1 or log2FC > 1. Using the ggVennDiagram” package in R, DEGs from both analyses were compared via Venn diagram and the percentage of Starvation DEGs found to be in common with SFN DEGs was calculated.

### Statistics

Statistical differences were measured with unpaired Student’s t-test or Analysis of Variance (ANOVA) that was appropriate for each data set and analysis. Normal distribution was verified by Shapiro–Wilk test and a Mann–Whitney nonparametric test was used when normal distribution was not present. Error bars represent standard deviations, and all experimental data derived from at least 3 independent replicates.

## Results

We previously reported that treating RPE-1 cells with SFN rapidly induced mitochondrial fusion and elongation (24), a phenotype similar to complete nutrient starvation (21). Deprivation of amino acids (AAs) or just glutamine was sufficient to induce fusion but removal of just serum or glucose caused mitochondrial fragmentation whereas coupling these reductions with AA deprivation exacerbated mitochondrial fusion (21). Functionally, the fused mitochondria were protected from starvation-induced degradation by autophagolysosomes (21). This shared mitochondrial fusion between SFN and nutrient deprivation prompted us to test the hypothesis that SFN phenocopies additional cellular responses to starvation.

### Effects of SFN on mitochondrial membrane potential, mass, matrix redox status, and mitophagy

**Figure 1:**
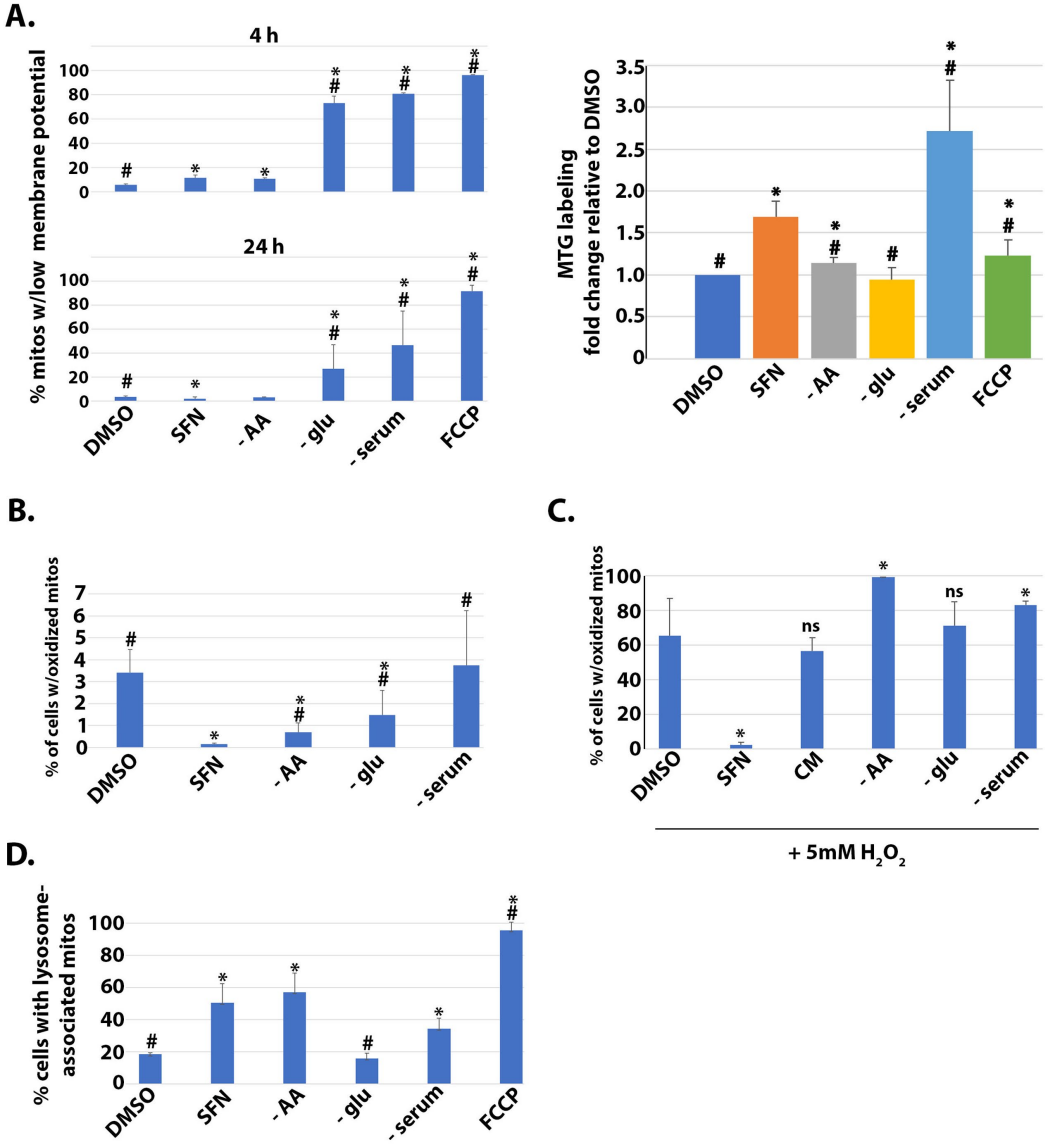
SFN promotes a reductive mitochondrial matrix environment, increased mitochondrial mass, mitophagy, and resistance to oxidative stress. **(A)** RPE-1 cells were treated with vehicle (DMSO), SFN, or culture media lacking AA (− AA), glucose (− glu), or serum (− serum) or with 5 µM FCCP for 4 (top graph) or 24 h (bottom and right graphs). Mitochondria were labeled with 200 nM MitoTracker Green (MTG) and 5 nM tetramethylrhodamine ethyl ester (TMRE) for 30 min. Cells were washed, trypsinized, analyzed by flow cytometry and graphed as the fraction of mitochondria with membrane potential loss or normalized to mitochondrial mass of DMSO-treated cells (right graph). **(B)** RPE-1 cells stably expressing mito-roGFP were treated as described for **(A)** for 4 h and then harvested for analysis by flow cytometry. Graph shows the fraction of cells containing mitochondria with oxidized matrices. **(C)** Mito-roGFP RPE-1 cells were treated as described for (B) with the addition of a complete media (CM) treatment before a 1 h challenge with 5 mM H_2_O_2_. Mitochondrial matrix oxidation was determined as for **(B)**. **(D)** RPE-1 cells stably expressing mtKeima and the E3 ligase parkin were treated as described for **(B)** and then analyzed by flow cytometry to determine the fraction of cells with lysosome-associated mitochondria. For **A**,**B**, and **D**, * indicates *p* < 0.05 compared to DMSO and # indicates *p* < 0.05 compared to SFN. For C, SFN is compared to DMSO whereas each nutrient deprivation is compared to CM. * indicates *p* < 0.05 for both comparisons and ‘ns’ = not significant.

We first compared the impacts of SFN versus different nutrient deprivations on mitochondrial mass and membrane potential. RPE-1 cells were labeled with MitoTracker Green (MTG) to mark total mitochondria and with tetramethylrhodamine ethyl ester (TMRE) to label mitochondria with intact membrane potential. Flow cytometry was used to quantify the uptake of both dyes, and FCCP treatment served as a positive control to show that the loss of mitochondrial membrane potential was readily detectable in this assay (Figure 1A, left graphs top and bottom). Membrane potential was measured at 4 h and 24 h post-treatment but mitochondrial mass was measured only at 24 h to provide sufficient time for mitochondrial biogenesis. SFN treatment caused a very modest but statistically significant loss of membrane potential at the 4 h time point but preserved mitochondrial membrane potential and increased mitochondrial mass after 24 h, most closely reflecting the mitochondrial response to AA deprivation (Figure 1A). In contrast, cells incubated in media lacking glucose showed reduced mitochondrial membrane potential, and serum deprivation increased mitochondrial mass but reduced mitochondrial membrane potential (Figure 1A).

In complementary experiments, we compared SFN treatment to nutrient deprivations to assess the extent of mitochondrial matrix oxidation, a proxy of mitochondrial redox status. These assays used RPE-1 cells stably expressing mito-roGFP, a mitochondrially-targeted GFP variant that harbors two redox-sensing cysteines (30). These cysteines modulate the excitation profile of the GFP, thereby allowing the redox state of the mitochondrial matrix to be quantified by measuring fluorescence intensity at the oxidizing and reducing excitation wavelengths (20). Four hour of SFN suppressed the fraction of mitochondria with an oxidized matrix (Figure 1B). Compared to DMSO-treated cells without starvation, both AA and glucose starvation also significantly decreased the percent of mitochondria with oxidized matrices but to a lesser extent than SFN. AA starvation looked most similar to SFN in this assay although all starvation conditions tested had statistically significant increases in oxidized mitochondrial matrices compared to SFN (Figure 1B).

This resistance to an oxidized mitochondrial matrix by SFN led us to test the hypothesis that the isothiocyanate could protect against mitochondrial oxidation induced by an exogenous oxidative challenge. Mito-roGFP RPE-1 cells were pre-treated for 4 h with SFN or DMSO followed by a 1 h exposure to 5 mM H_2_O_2_ to induce mitochondrial oxidation. Cells subjected to various nutrient starvations were likewise challenged with H_2_O_2_ (Figure 1C). In contrast to cells deprived of nutrients, versus complete media (CM), SFN prevented mitochondrial oxidation induced by H_2_O_2_ (Figure 1C). We next examined if SFN impacted homeostatic levels of mitochondria being targeted to lysosomes for mitophagy using RPE-1 cells stably expressing mitochondria-targeted Keima (mtKeima) and the parkin E3 ligase. mtKeima is a pH-dependent fluorescent protein containing the mitochondrial targeting sequence from COX VIII. mtKeima excites at 440 nm when mitochondria are in the cytoplasm and at 586 nm when mitochondria have been engulfed by lysosomes. The emission wavelength for mtKeima is ~620 nm and is independent of pH (31). As homeostatic mitophagy is minimal in unchallenged RPE-1 cells, stable over-expression of parkin accelerates mitophagy (31) to enable the evaluation and comparison of acute treatments (i.e., 4 h). FCCP treatment validated the sensitivity of the assay for tracking mitochondrial turnover by mitophagy in the time frame tested (Figure 1D). SFN, AA starvation, and serum deprivation all individually increased the percent of cells undergoing mitophagy whereas glucose deprivation was comparable to unstarved, DMSO-treated cells (Figure 1D). Together, these data show that SFN preserves mitochondrial membrane potential, limits mitochondrial matrix oxidation, and promotes homeostatic mitophagic flux (i.e., mitophagy and biogenesis), thereby mirroring aspects of nutrient deprivation, with the most similarities to AA starvation.

### Impacts of SFN on signaling through mTOR

**Figure 2:**
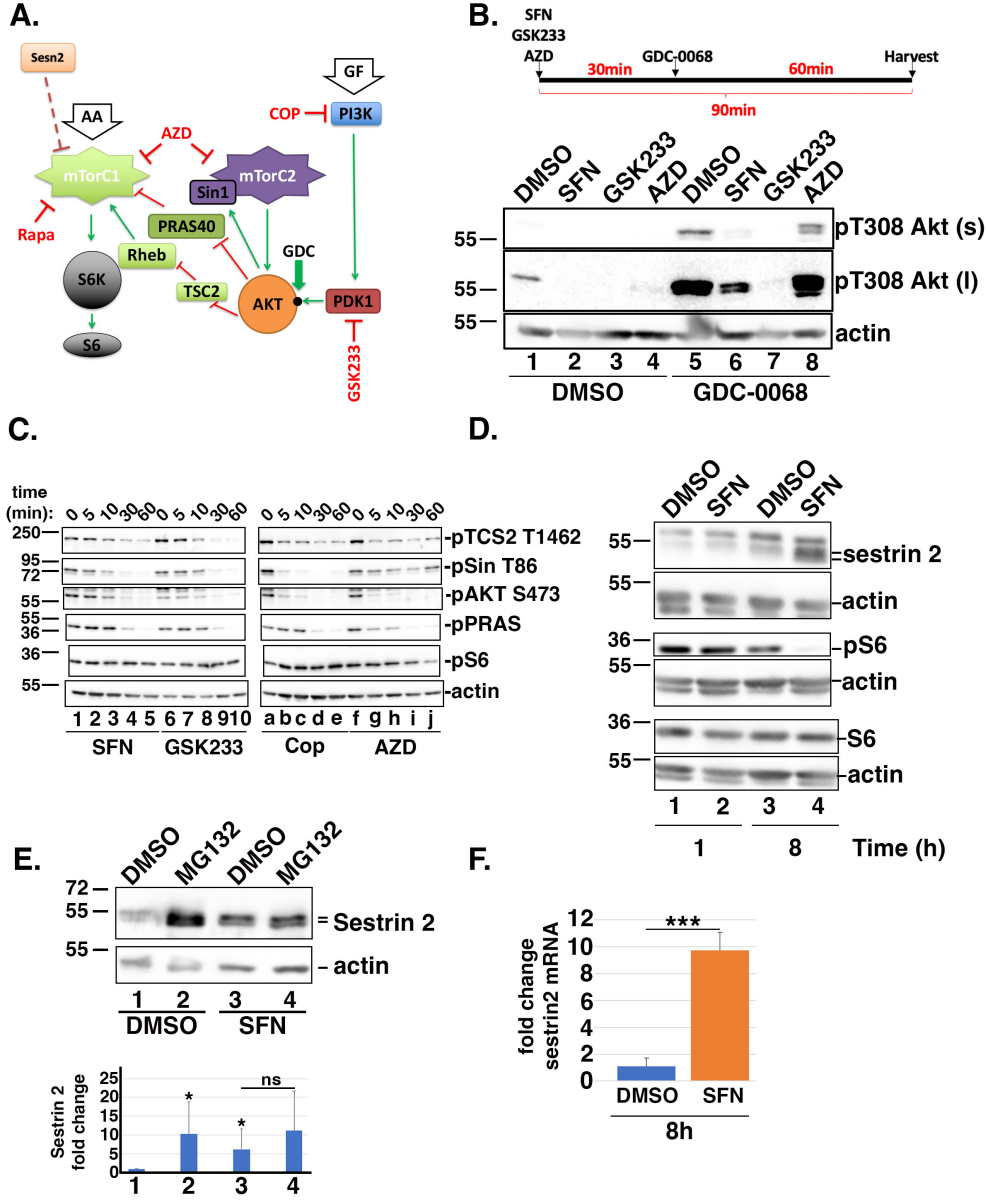
SFN disrupts insulin and mTORC1/2 signaling. **(A)** Cartoon highlighting the proteins and phosphorylation events analyzed within the insulin and mTORC1/2 signaling cascades. Green arrows indicate activation and red ‘T’ symbols indicate inhibition. Pharmacological inhibitors are denoted in red text. Black dot on AKT marks the threonine 308 (T308) phosphoacceptor. AA = amino acids; GF = growth factors; Sesn2 = Sestrin 2; S6K = S6 kinase. **(B)** Schematic of the experimental design. RPE-1 cells were treated for 90 min with DMSO, SFN, 1 µM GSK233 or 250 nM AZD and co-treated with DMSO (lanes 1–4) or with 5 µM GDC-0068 (lanes 5–8) for the last 60 min of the 90 min incubation. Lysates were analyzed by Western blotting with antibodies against phosphorylated Akt on T308 (top and middle blots) and actin (bottom blot). ‘s’ and ‘l’ denote short and long exposures, respectively. * indicates p < 0.05 compared to DMSO/DMSO (lane 1) and ‘ns’ on graph indicates no significance between lanes 3 and 4. **(C)** RPE-1 cells were treated with SFN, GSK233, Cop, or AZD for the indicated times. Lysates were probed with antibodies specific for the phosphorylated forms of TSC2 on threonine 1,462 (pTSC2 T1462), Sin on threonine 86 (pSin T86), Akt on serine 473 (pAKT S473), PRAS on threonine 246 (pPRAS), and S6 on serines 235/236 (pS6) as well as actin. **(D)** RPE-1 cells were treated with DMSO or SFN for 1 or 8 h and harvested for Western blot analysis with the indicated antibodies. **(E)** RPE-1 cells were treated with DMSO or SFN with or without 5 µM MG132 for 8 h. Sestrin 2 protein expression was analyzed by Western blotting and graph shows quantification of Sestrin 2 protein levels from 5 independent experiments. Numbers below graph correspond to lane numbers from a representative Western blot. **(F)** qPCR of *sestrin 2* mRNA expression levels following 8 h of DMSO or SFN. For all western blots, the migration of molecular weight markers (in kDa) is indicated to the left of each blot. Blots in B-D are representative of 3–5 independent experiments, and all graphed data were compiled from ≥3 independent experiments with DMSO set to a value of 1.0. *** indicates *p* < 0.001.

Nutrient signaling through the mechanistic target of rapamycin (mTOR) involves two integrated kinase hubs, mTORC1 and mTORC2. These complexes function as primary sensors of amino acids (mTORC1) and growth factors (mTORC2) (reviewed in (32)). In time-course experiments analyzing the acute effects of SFN on nutrient sensing, we tracked the phosphorylation of multiple proteins in the insulin signaling cascade with a focus on phosphatidylinositol 3-kinase (PI-3 K), Akt, and downstream substrates and effectors. These experiments compared SFN to the pharmacological inhibitors of insulin signaling GSK2334470 (GSK233), AZD8055 (AZD), and copanlisib (Cop). Targets of each inhibitor are diagrammed in Figure 2A. GSK233 inhibits phosphoinositide-dependent kinase-1 (PDK1), the kinase that activates Akt via phosphorylation of threonine 308 (T308), whereas AZD inhibits both mTORC1 and mTORC2, and Cop inhibits PI-3 K.

Compared to DMSO, phosphorylation of T308 on Akt was decreased by SFN, GSK233, and AZD (Figure 2B, lanes 1–4). To facilitate visualizing T308 phosphorylation, the signal was amplified by co-treating cells for the last 60 min of the 90 min incubation with GDC-0068 (GDC), an agent that blocks phosphatase access to T308 (33). This signal enhancement confirmed that SFN reduced T308 phosphorylation (Figure 2B, lane 5 versus 6) albeit to a lesser extent than the PDK1 inhibitor, GSK233 (Figure 2B, lane 6 versus 7), and that AZD altered the pattern of Akt phosphorylation (Figure 2B, lane 5 versus 8). T308 phosphorylation primes Akt to phosphorylate threonine 86 (T86) of Sin, an activating subunit of mTORC2. In turn, activated mTORC2 feeds back to phosphorylate Akt on serine 473 (S473) to generate fully activated Akt, which then phosphorylates and inhibits the downstream effectors PRAS and TSC2 (34, 35) (Figure 2A). In kinetic studies with homeostatic phosphorylation levels represented by time point ‘0’, the reduction by SFN of the priming T308 phosphorylation on Akt (Figure 2B) led to reduced phosphorylation of T86 on Sin and S473 on Akt by mTORC2 (Figure 2C, lanes 1–5). Consistent with dampening Akt activation, SFN reduced phosphorylation of the downstream substrates PRAS and TSC2 over the course of 60 min (Figure 2C, lanes 1–5). Notably, TSC2 is part of a complex that inhibits mTORC1, and is a phosphorylation substrate of AMPK (36). When compared to pharmacological inhibitors, the kinetics and specificity of phosphorylation loss mediated by SFN for each protein analyzed most closely approximated those observed when PDK1 was pharmacologically inhibited by GSK233 (Figure 2C, lanes 1–5 compared to 6–10). PI-3 K is the kinase directly upstream of PDK1 and Cop inhibits PI-3 K as well as the generation of PIP_3_, which activates mTORC2 through direct binding to the PH-domain of Sin (37). Therefore, in cells treated with Cop, the loss of pSin T86 and pAKT S473 occurred within 5 min (Figure 2C, lanes a-e). Likewise, because AZD inhibits mTORC2, pSin T86 is largely preserved while loss of pAKT S473 also occurs within the first 5 min and pS6 decreased by 1 h (Figure 2C, lanes f-j). Together, these data show that SFN elicits a temporal phosphorylation pattern resembling the PDK1 inhibitor, GSK233. In contrast to the relatively rapid inhibition of mTORC2 signaling by SFN, phosphorylation of S6 (pS6) downstream of mTORC1 was unchanged during the first 60 min of treatment (Figure 2C, lanes 1 and 5). However, 8 h of SFN markedly reduced pS6 levels with a concomitant increase in the mTORC1 inhibitor, Sestrin 2. (Figure 2D, lane 4). The elevation of Sestrin 2 by SFN is mediated in part by stability/turnover. Sestrin 2 is stabilized by SFN (Figure 2E, lane 1 versus 3) and by MG132 in the absence of SFN (Figure 2E, lane 1 versus 2) but inhibition of the proteasome in the presence of SFN did not further increase Sestrin 2 levels (Figure 2E, lane 3 versus 4), indicating that the phytochemical limits the proteasomal degradation of Sestrin 2. Elevated Sestrin 2 is also mediated at the transcriptional level as SFN increased *Sestrin 2* mRNA by ~10-fold (Figure 2F).

### Effects of SFN on autophagic flux and lysosomes

**Figure 3:**
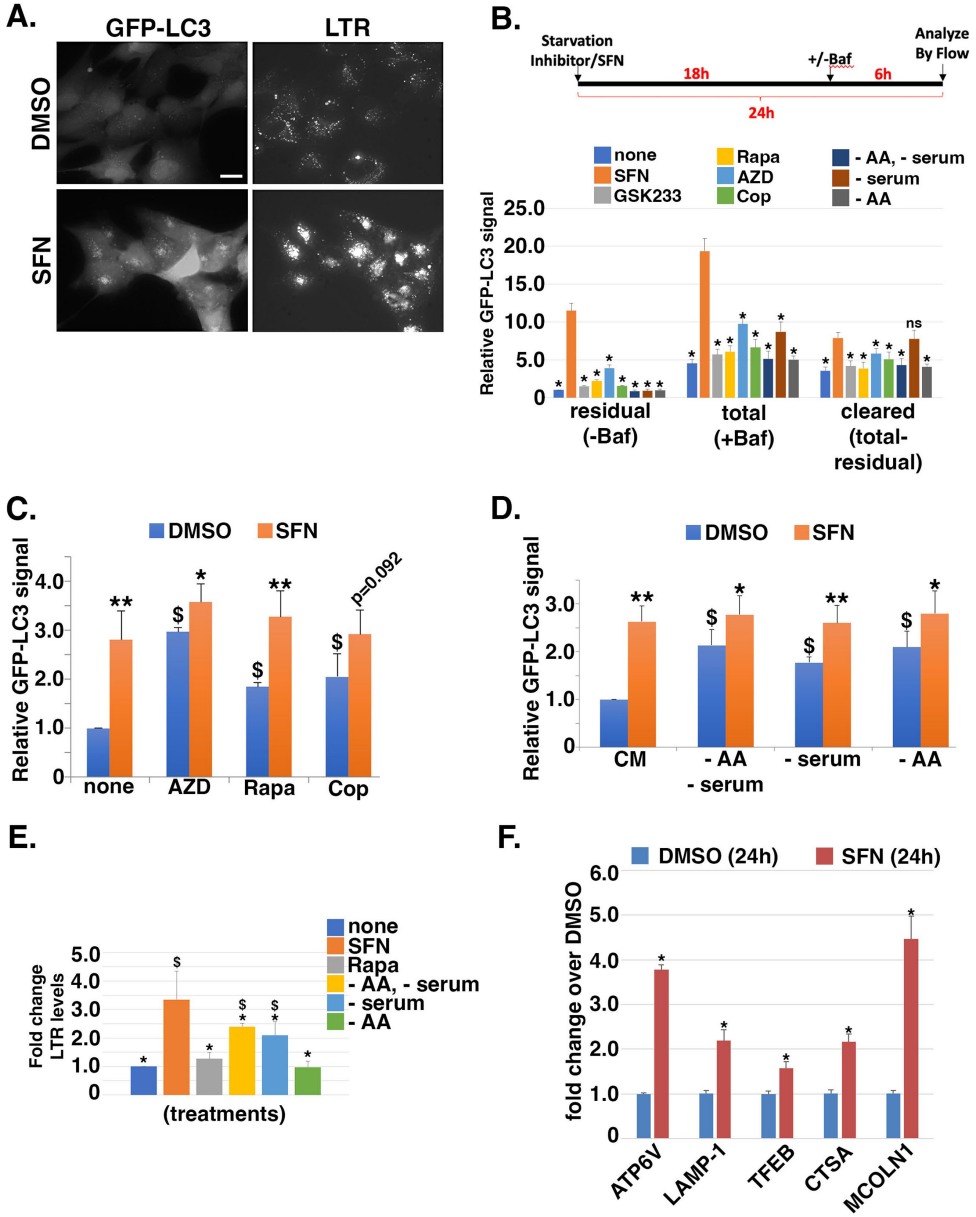
SFN is a potent inducer of autophagy and increases lysosomal mass. **(A)** RPE-1 cells stably expressing GFP-LC3 were treated with DMSO or SFN for 8 h and 50 nM LysoTracker Red™ (LTR) was added during the final 30 min. Figure shows representative live-cell photomicrographs. Size bar = 10 microns. **(B)** Schematic of the experimental design. RPE-1 cells stably expressing GFP-LC3 were treated with vehicle (none), SFN, GSK233, Rapa, AZD, Cop or media lacking amino acids and serum (−AA/−serum), lacking just serum (− serum), or lacking just AA (− AA) for 24 h. Vehicle (-Baf) or 160 µM Bafilomycin (+Baf) were added for the final 6 h of the 24 h incubation. Cells were trypsinized, resuspended in 0.05% (w/v) saponin/PBS, washed, and GFP-LC3 fluorescence intensity was analyzed by flow cytometry. Data graphed as GFP-LC3 fluorescence intensity relative to vehicle for residual (–Baf), total (+Baf) and cleared (total-residual). * indicates *p* ≤ 0.05 for comparison versus SFN (orange bars). **(C,D)** GFP-LC3-expressing RPE-1 cells were co-treated with the indicated autophagy inducers **(C)** or nutrient deprivations **(D)** plus DMSO (blue bars) or SFN (orange bars) for 24 h. Cells were processed and analyzed by flow cytometry as described for **(B)**. Data graphed as GFP-LC3 fluorescence intensity relative to none + DMSO **(C)** or CM + DMSO **(D)**. **(E)** RPE-1 cells were treated with SFN, 10 nM Rapa, or media lacking the indicated nutrients (as in B) for 16 h, stained with 50 nM LTR for 30 min, trypsinized, and analyzed by flow cytometry. Data graphed as fold change in fluorescence intensity compared to no treatment (none). **(F)** RPE-1 cells were treated with DMSO or SFN for 24 h before RNA isolation and qPCR using primers against the indicated CLEAR network gene products. Results graphed as fold change in mRNA levels compared to DMSO. For **(C,D)**, asterisks indicate significance between each paired set of blue and orange bars, respectively; *p ≤ 0.05, ** *p* ≤ 0.01, and $ indicates significance compared to ‘none’ + DMSO for **(C)** or CM + DMSO for **(D)**; *p* ≤ 0.05.

We next tested the hypothesis that SFN would activate autophagy since suppression of mTORC1/2 relieves autophagy inhibition to promote the salvaging of cellular bioenergetic substrates and building blocks (38). In addition, SFN has been reported to both activate [e.g., (39, 40)] and inhibit [e.g., (41)] autophagy based on Western blotting for microtubule-associated protein 1A/1B-light chain 3 (LC3) to measure the lipidated and unlipidated forms (LC3-I and LC3-II, respectively). Increased LC3-II levels are typically interpreted as a proxy of elevated autophagic flux. However, because LC3-II accumulation can occur through either an increase in autophagic flux or conversely, a block in autophagy progression downstream of autophagosome formation, we used RPE-1 cells stably expressing GFP-tagged LC3 (GFP-LC3) (25) in combination with bafilomycin A1 (Baf) and flow cytometry to determine the consequences of SFN on autophagic flux. Baf is a macrolide antibiotic that blocks the autophagy pathway by preventing fusion of autophagosomes to lysosomes and by inhibiting the vacuolar H^+^ ATPase to prevent the acidification of lysosomes and protein degradation (42). Eight hours of SFN treatment led to increased GFP-LC3 puncta as well as increased LysoTracker Red™ (LTR)-positive puncta, a marker of lysosomes (Figure 3A). To further analyze autophagic flux, RPE-1 cells stably expressing GFP-LC3 were treated with SFN, the indicated pharmacological treatments, or deprived of different nutrients for 24 h. Parallel wells of cells were co-treated with Baf during the final 6 h of the 24 h incubation. Prior to quantifying GFP-LC3 levels by flow cytometry, cells were permeabilized with saponin to leak out soluble GFP-LC3 not incorporated into autophagic vesicles. As Baf inhibits the degradation of autophagic cargoes, the amount of GFP-LC3 in cells incubated with Baf represents the “total” amount of autophagic traffic that entered the system during the final 6 h of the experiment. GFP-LC3 levels in cells not treated with Baf represent “residual” traffic actively transiting through the autophagy system, and the difference (i.e., total – residual) represents the amount of “cleared” autophagic traffic. Compared to established inducers of autophagy including rapamycin (Rapa), inhibitors of insulin signaling (GSK233, AZD, Cop), and nutrient deprivations, SFN treatment yielded greater amounts of total and residual GFP-LC3 autophagic vesicles (Figure 3B). With the exception of serum deprivation (− serum), SFN induced greater clearance of autophagic vesicles compared to the other treatments (Figure 3B).

The amplification of autophagic flux by SFN was further corroborated by testing whether combining the phytochemical with pharmacological inducers of autophagy (Figure 3C) or with nutrient deprivations that induce autophagy (Figure 3D) would further augment vesicular GFP-LC3 beyond the levels reached by each individual treatment. RPE-1 cells stably expressing GFP-LC3 were incubated for 24 h with inducers of autophagy and compared to the same treatments combined with SFN. Remarkably, co-treatment with SFN and pharmacological inducers of autophagy elevated GFP-LC3 levels with the exception of Cop, which approached significance (*p* = 0.092; Figure 3C). Moreover, depriving cells of serum and/or AA induced autophagy and SFN also amplified these inductions (Figure 3D).

We next tested if the increased autophagic flux induced by SFN was accompanied by increased cellular lysosomal content, as indicated by fluorescence microscopy of LTR-labeled cells (Figure 3A). We quantified lysosomal content by LTR uptake using flow cytometry, comparing SFN to nutrient deprivations and to Rapa (Figure 3E). Cells were treated with SFN, Rapa, or starved of specific nutrients overnight and then stained with LTR. Notably, neither Rapa nor AA starvation increased lysosomal content (Figure 3E gray and green bars, respectively) indicating that lysosomal biogenesis was responsive to the inhibition of insulin signaling but not inhibition of mTORC1 by Rapa or AA deprivation in this assay. SFN robustly increased lysosomal content, consistent with the phytochemical inducing the transcription factor EB (TFEB)-directed CLEAR transcriptional program downstream of mTOR, as recently reported (43). Using qPCR, we confirmed that 24 h of treatment with SFN induced the expression of canonical CLEAR target genes (*ATP6V*, *LAMP1*, *TFEB*, *CTSA*, and *MCOLN1*) by 1.5-to 4.5-fold over vehicle treatment (Figure 3F).

### Impacts of SFN on glycolysis, the TCA cycle, and the hexosamine biosynthetic pathway

**Figure 4:**
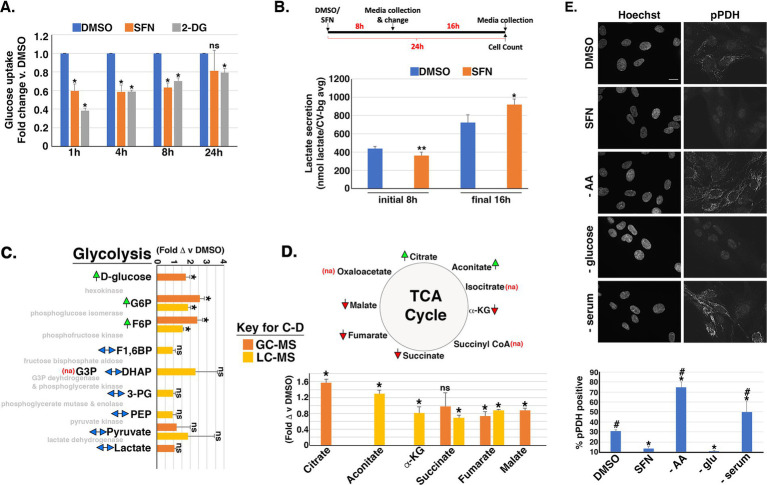
SFN impacts flux through glycolysis and the TCA cycle. **(A)** RPE-1 cells were treated with DMSO, SFN or 5 mM 2-DG for the indicated times followed by glucose uptake measurements using the Dojindo Green Probe per manufacturer’s instructions. Glucose uptake was graphed as fold change versus DMSO treatment. **(B)** Schematic of the experimental design. RPE-1 cells were treated with DMSO or SFN for 8 h after which media was collected (initial 8 h measurement) and replaced with fresh media containing DMSO or SFN for an additional 16 h (for a total of 24 h treatment). Media was again collected for the final 16 h measurement. Media from the initial 8 h incubation and from the final 16 h incubation were separately analyzed with the L-Lactate Assay Kit I (Eton Biosciences, Inc.) and normalized to cell number with data graphed as lactate secretion per normalized cell number. **(C,D)** RPE-1 cells were treated with DMSO or SFN for 4 h and then analyzed by GC–MS (orange bars) and/or LC–MS (yellow bars) for glycolytic intermediates **(C)** or TCA metabolites **(D)**. Green arrows indicate elevation of the metabolite by SFN, red arrows indicate suppression of the metabolite by SFN, and two-headed blue arrows indicate no change in the metabolite. ‘na’ denotes that the metabolite was not assayed. The enzymes that mediate each step of glycolysis are shown in light gray text for reference **(C)**. Abbreviations: Glucose-6-phosphate (G6P), fructose-6-phosphate (F6P), fructose-1,6-bisphosphate (F1,6P), Glyceraldehyde-3-phosphate (G3P), dihydroacetone-phosphate (DHAP), 3-phosphoglycerate (3PG), Phosphoenolpyruvate (PEP), α-ketoglutarate (α-KG). **(E)** RPE-1 cells were treated as indicated for 4 h and labeled with the DNA counterstain Hoechst and an antibody to phosphorylated pyruvate dehydrogenase (pPDH). Size bar represents 10 microns. Graph of the fraction of pPDH-positive cells counted as a function of treatment with DMSO, SFN, or the indicated nutrient deprivations. All graphs compiled from ≥3 independent experiments. For **(A,C,D)**, asterisks indicate statistical significance (*p* < 0.05) versus DMSO for each time point; ‘ns’ denotes not significant. For B, comparisons are between DMSO and SFN for each time point with * indicating *p* ≤ 0.01 and ** indicating *p* ≤ 0.001. For **E**, asterisks indicate statistical significance (*p* < 0.05) compared to DMSO, # indicates statistical significance (*p* < 0.05) compared to SFN, and ‘ns’ denotes not significant.

We next interrogated the time-dependent impacts of SFN on glucose uptake and metabolism based on work showing SFN, through its induction of Nrf2 transcriptional activity, induces genes encoding the rate limiting enzymes of pathways that shunt glucose out of glycolysis (44). We quantified glucose uptake using flow cytometry to measure the intracellular accumulation of a fluorescent glucose analog added to the media. Glucose uptake was reduced by SFN at 1 h, 4 h, and 8 h post-treatment similarly to 2-deoxy-glucose (2-DG), an established inhibitor of glucose uptake (Figure 4A). This reduced glucose uptake correlated with decreased extracellular lactate levels during the initial 8 h of SFN exposure (Figure 4B). However, glucose uptake recovered within 24 h of SFN treatment, with levels comparable to DMSO-treated cells (Figure 4A), and lactate secretion rebounded accordingly (Figure 4B, final 16 h). Complementary metabolomic analysis of glycolytic and tricarboxylic acid (TCA) cycle intermediates showed that 4 h of SFN led to the accumulation of intracellular glucose and the two downstream glycolytic intermediates, glucose-6-phosphate (G6P) and fructose-6-phosphate (F6P) (Figure 4C). Other assayed glycolytic intermediates were unchanged. TCA metabolite analysis revealed increased citrate and aconitate whereas a-ketoglutarate, succinate, fumarate and malate were all decreased (Figure 4D).

Concomitant with these effects on glycolysis and the TCA cycle, SFN reduced phosphorylation of PDH (Figure 4E), the mitochondrial enzyme that converts pyruvate to acetyl-CoA for the TCA cycle. Phosphorylation of PDH (pPDH) inactivates the enzyme and dephosphorylation converts PDH to an active form (45, 46). Immunofluorescence assays with an antibody recognizing Ser293 phosphorylation of PDH (pPDH), a proxy of PDH activity (47, 48), revealed that 4 h of SFN treatment robustly reduced the percentage of cells with mitochondria-associated pPDH (Figure 4E). AA deprivation or serum starvation both increased pPDH whereas glucose starvation reduced pPDH (Figure 4E). Thus, SFN most closely mimicked glucose starvation in this assay.

**Figure 5:**
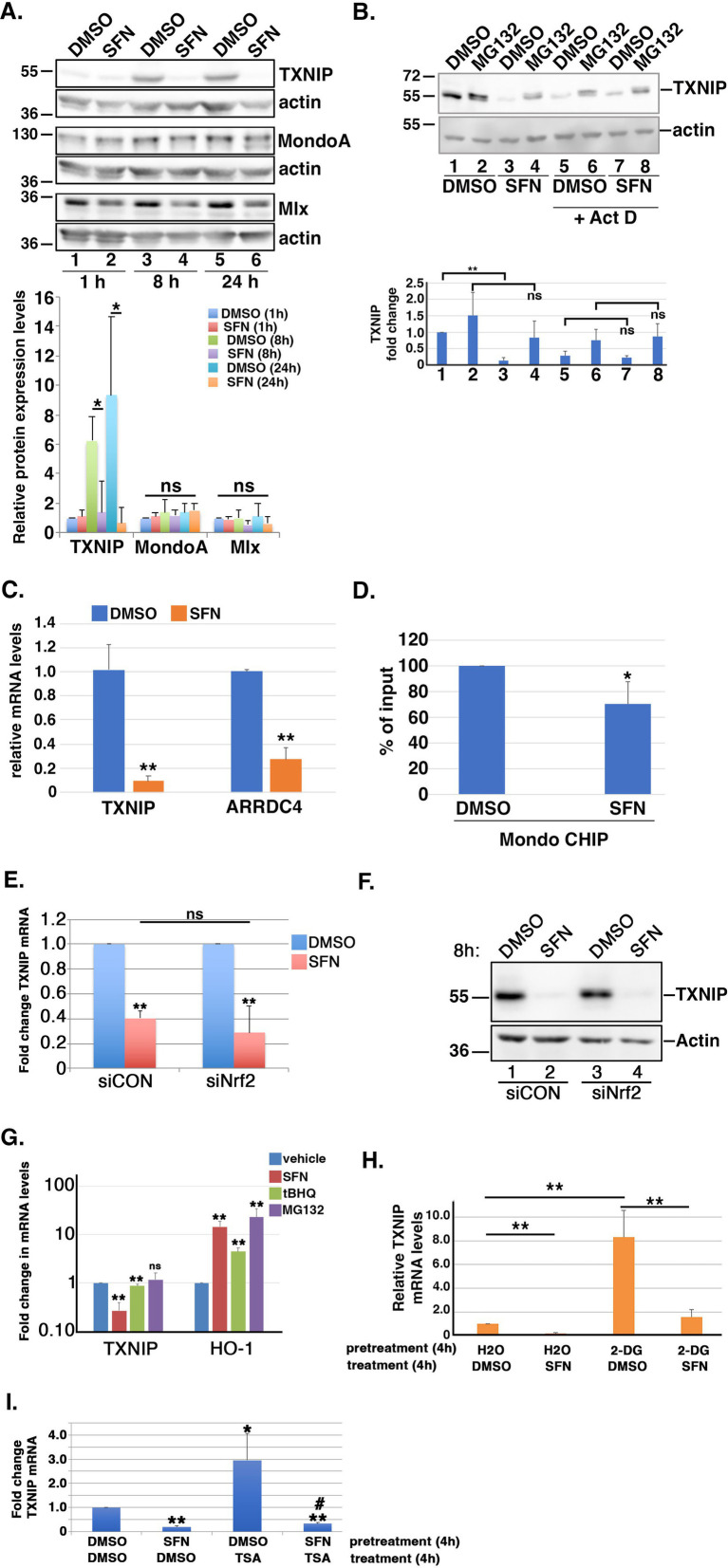
SFN suppresses TXNIP expression. **(A)** RPE-1 cells were treated with DMSO or SFN for the indicated times and lysates were run in triplicate for analysis by Western blotting with antibodies against TXNIP, MondoA, Mlx, and actin. Graph below blots shows quantified levels of TXNIP, MondoA, and Mlx (relative to 1 h of DMSO treatment) from >3 independent experiments. **(B)** RPE-1 cells were treated with DMSO or SFN with or without 5 µM MG132 for 8 h. Cells in lanes 5–8 were also treated with 1 µM Act D. TXNIP protein expression was analyzed by Western blotting and graph shows quantification of TXNIP protein levels. Numbers below graph correspond to lane numbers from Western blot. **(C)** RPE-1 cells treated with DMSO or SFN for 4 h were analyzed for *TXNIP* and *ARRDC4* mRNA levels by qPCR. Graph of qPCR data shows change relative to DMSO treatment. **(D)** Chromatin from RPE-1 cells treated with DMSO or SFN for 4 h was analyzed by CHIP with primers specific to the promoter of TXNIP and graphed as fraction of DMSO treatment. **(E,F)** RPE-1 cells were treated for 2 days with control siRNA (siCON) or siRNA targeting Nrf2 (siNrf2) and then exposed to DMSO or SFN for 8 h. Cells were then harvested to measure *TXNIP* mRNA levels by qPCR **(E)** and TXNIP protein expression by Western blotting **(F)**. The migration of molecular weight markers (in kDa) is indicated to the left of the blots. **(G)** RPE-1 cells treated with DMSO, SFN, 5 µM tBHQ or 5 µM MG132 for 8 h were harvested to measure mRNA levels of *TXNIP* and *HO-1* relative to vehicle treatment. **(H)** RPE-1 cells were pre-treated for 4 h with either vehicle (H_2_O) or 5 mM 2-DG followed by an additional 4 h with either DMSO or SFN and then were harvested to measure *TXNIP* mRNA levels by qPCR. Graph of *TXNIP* mRNA expression levels relative to H_2_O + DMSO. **(I)** RPE-1 cells were pre-treated for 4 h with either DMSO or SFN followed by an additional 4 h with either DMSO or 100 nM TSA prior to being harvested for qPCR to measure *TXNIP* mRNA levels. Asterisks indicate statistical significance compared to DMSO/DMSO and # indicates statistical significance (*p* < 0.05) compared to DMSO/TSA. All graphed data compiled from ≥3 independent experiments. * indicates *p* < 0.05, ** indicates *p* < 0.01 and ‘ns’ denotes not significant.

The accumulation of G6P, the first oxidation product of glycolysis, can elevate the expression of thioredoxin (TXN)-interacting protein (TXNIP), a central regulator of glucose entry into cells (49–53). By modulating glucose transporter (GLUT) endocytosis, increased levels of TXNIP reduce cellular glucose uptake whereas decreased TXNIP abundance promotes glucose uptake into cells [e.g., (49, 52)]. Based on SFN treatment acutely reducing glucose uptake but then restoring it by 24 h (Figure 4A), we tested the hypothesis that this restoration was mediated by reducing TXNIP despite increased G6P levels. After confirming that *TXNIP* mRNA and protein expression are glucose-responsive and dependent in RPE-1 cells (Supplementary Figure S1A), we examined TXNIP levels by Western blotting lysates from RPE-1 cells treated with the phytochemical for different lengths of time. Compared to vehicle-treated cells, 8 h and 24 h of SFN treatment dramatically reduced TXNIP protein expression (Figure 5A, lanes 3 versus 4 and 5 versus 6). Notably, the elevation of TXNIP at 8 and 24 h in the DMSO samples compared to the 1 h time point is consistent with glucose being taken up by the RPE-1 cells initially after replating into fresh media followed by an increase in TXNIP to restrict further uptake at the later time points (Figure 5A, lanes 3 and 5). Co-treatment with the proteasome inhibitor, MG132, stabilized TXNIP indicating that proteasome-mediated degradation of this protein was not adversely impacted by SFN (Figure 5B, lanes 3 versus 4) (49, 51). In contrast, co-administration of SFN with the global transcription inhibitor, actinomycin D (Act D), normalized TXNIP expression between DMSO and SFN in the presence and absence of MG132 (Figure 5B, lanes 5 versus 7 and lanes 6 versus 8), indicating that the SFN-mediated decrease in TXNIP occurs at the transcriptional level. qPCR analysis confirmed that 4 h of SFN robustly decreased *TXNIP* mRNA expression as well as the mRNA levels of the TXNIP paralogue, *ARRDC4* (Figure 5C).

After ruling out that SFN was not accelerating the degradation of *TXNIP* mRNA (Supplementary Figure S1B), we examined the glucose-dependent transcription complex MondoA/Mlx. This heterodimeric transcription factor mediates *TXNIP* and *ARRDC4* mRNA expression by binding a carbohydrate response element within their respective promoters (52). 1, 8, or 24 h of SFN treatment did not statistically significantly decrease MondoA or Mlx protein levels (Figure 5A blots and graph). Endogenous protein co-immunoprecipitations showed that SFN also does not disrupt complex formation between MondoA and Mlx (Supplementary Figure S1C). Chromatin immunoprecipitation (CHIP) assays however revealed that SFN reduced MondoA residency on the *TXNIP* promoter by ~30% (Figure 5D).

As Nrf2 can bind to the *TXNIP* promoter (54), we next tested the hypothesis that SFN-mediated suppression of *TXNIP* mRNA and protein levels is Nrf2 dependent. After confirming the efficiency of *Nrf2* mRNA knockdown by siRNA using qPCR (Supplementary Figure S1D), we found that *TXNIP* mRNA and protein suppression by SFN were comparable between control (siCON) and Nrf2 (siNrf2) knockdown cells (Figures 5E, F, respectively). Additionally, stabilization of Nrf2 by other activators had only a very modest or no effect on *TXNIP* mRNA levels despite robustly inducing the Nrf2 target gene, heme-oxygenase 1 (*HO-1*) (Figure 5G). These data show that the suppression of TXNIP mRNA and protein expression by SFN is independent of Nrf2 induction.

Ayer and colleagues demonstrated that 2-DG induces the Mondo/Mlx transcriptional activation of *TXNIP* (52). This glucose derivative is phosphorylated by hexokinase to produce 2-DG-6-phosphate, a product that cannot be further processed for glucose oxidation and is a relatively poor substrate for glucose-6-phosphate dehydrogenase (G6PDH) (55). We therefore tested the hypothesis that SFN could suppress 2-DG-mediated TXNIP induction. Four hours of 2-DG followed by 4 h of DMSO elevated *TXNIP* mRNA expression ~8-fold but 4 h of SFN treatment suppressed this *TXNIP* induction (Figure 5H). Likewise, *TXNIP* transcription is also increased by treatment with Trichostatin A (TSA) (Figure 5I), a Class I/II histone deacetylase inhibitor (56). Pretreatment for 4 h with SFN prevented the TSA-induced transcriptional increase of *TXNIP* (Figure 5I). These data demonstrate that SFN suppresses TXNIP mRNA induction and does so independently of Nrf2 activation.

**Figure 6:**
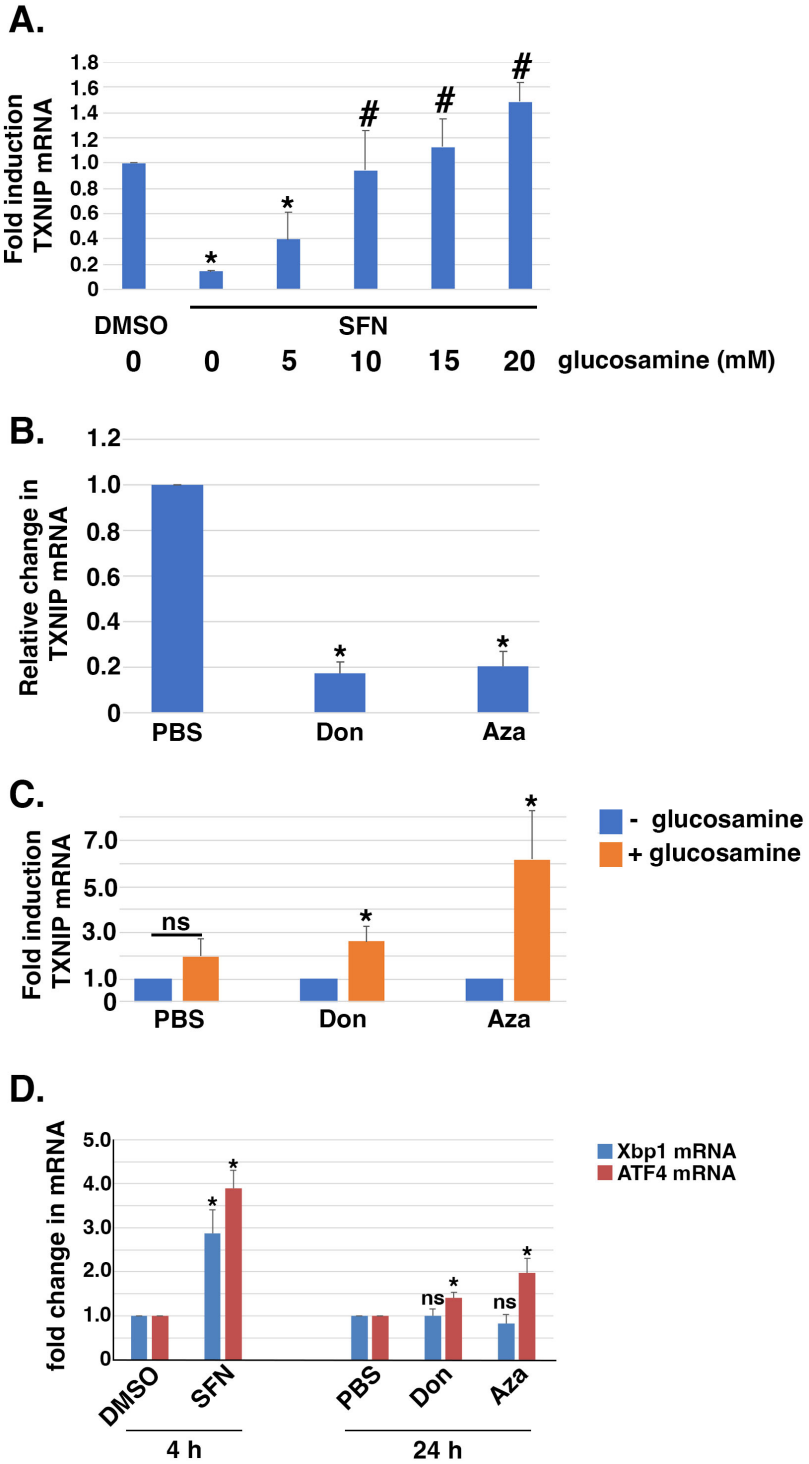
Exogneous glucosamine rescues SFN-mediated suppression of *TXNIP* mRNA expression. **(A)** RPE-1 cells treated with DMSO or SFN +/− increasing amounts of glucosamine for 4 h were harvested to measure *TXNIP* mRNA levels by qPCR. **(B)** RPE-1 cells were incubated with PBS or 100 µM 6-diazo-5-oxo-L-norleucine (Don) or 2.5 µM azaserine (Aza) for 24 h and then harvested for qPCR to measure *TXNIP* mRNA expression levels. **(C)** RPE-1 cells were incubated with either vehicle (PBS), Don, or Aza in the absence (blue bars) or presence (orange bars) of 15 mM glucosamine for 24 h prior to being harvested for qPCR to quantify *TXNIP* mRNA levels. **(D)** RPE-1 cells were treated for 4 h with DMSO or SFN or with PBS, Don, or Aza for 24 h and then harvested to measure mRNA expression of *Xbp1* (blue bars) or *ATF4* (red bars) by qPCR. All graphs compiled from 3 independent experiments with asterisks indicating statistical significance (*p* < 0.05) and ‘ns’ denoting not significant. For **(A)**, asterisks denote statistical significance compared to DMSO and # denotes significance compared to SFN without glucosamine supplementation. For **(D)**, statistical significance was determined by comparing SFN to DMSO and by comparing Don and Aza to PBS.

The hexosamine biosynthetic pathway (HBP) diverts F6P from glycolysis into an anabolic pathway for *de novo* generation of uridine diphosphate-N-acetyl glucosamine (UDP-GlcNAc), the substrate transferred to recipient proteins for N-and O-linked glycosylation reactions (reviewed in (57)). Notably, HBP activation can increase TXNIP mRNA expression (58). Because SFN treatment increased F6P levels (Figure 4C), we tested the hypothesis that the phytochemical inhibits glutamine F6P amidotransferase (GFAT), the first and rate-limiting enzyme of the HBP that converts F6P to glucosamine-6-phosphate. Consistent with this hypothesis, the suppression of *TXNIP* mRNA expression by SFN was reversed by supplying cells with exogenous glucosamine and this rescue was dose-dependent (Figure 6A). Glucosamine is phosphorylated by hexokinase to produce glucosamine-6-phosphate, the product produced by GFAT from F6P and glutamine. Thus, providing glucosamine to cells bypasses GFAT inhibition and increases *TXNIP* mRNA expression. This interpretation was independently confirmed using two GFAT pharmacological inhibitors, 6-diazo-5-oxo-L-norleucine (Don) and azaserine (Aza). Inhibiting GFAT with Don or Aza suppressed *TXNIP* mRNA expression ~ 80% (Figure 6B), and exogenous glucosamine likewise overcame this GFAT block leading to elevated *TXNIP* (Figure 6C). Consistent with the HBP being integrated with the unfolded protein response (UPR) and ER stress (59), 4 h of SFN upregulated the mRNA expression of the UPR-and ER stress responsive transcription factors XBP1 and ATF4 (Figure 6D). In contrast, inhibition of GFAT with either Don or Aza failed to increase *XBP1* mRNA expression and only modestly increased *ATF4* mRNA expression. These data demonstrate that the suppression of TXNIP mRNA expression by SFN likely results from inhibiting GFAT.

### Acute effects of SFN on the transcriptional landscape

**Figure 7:**
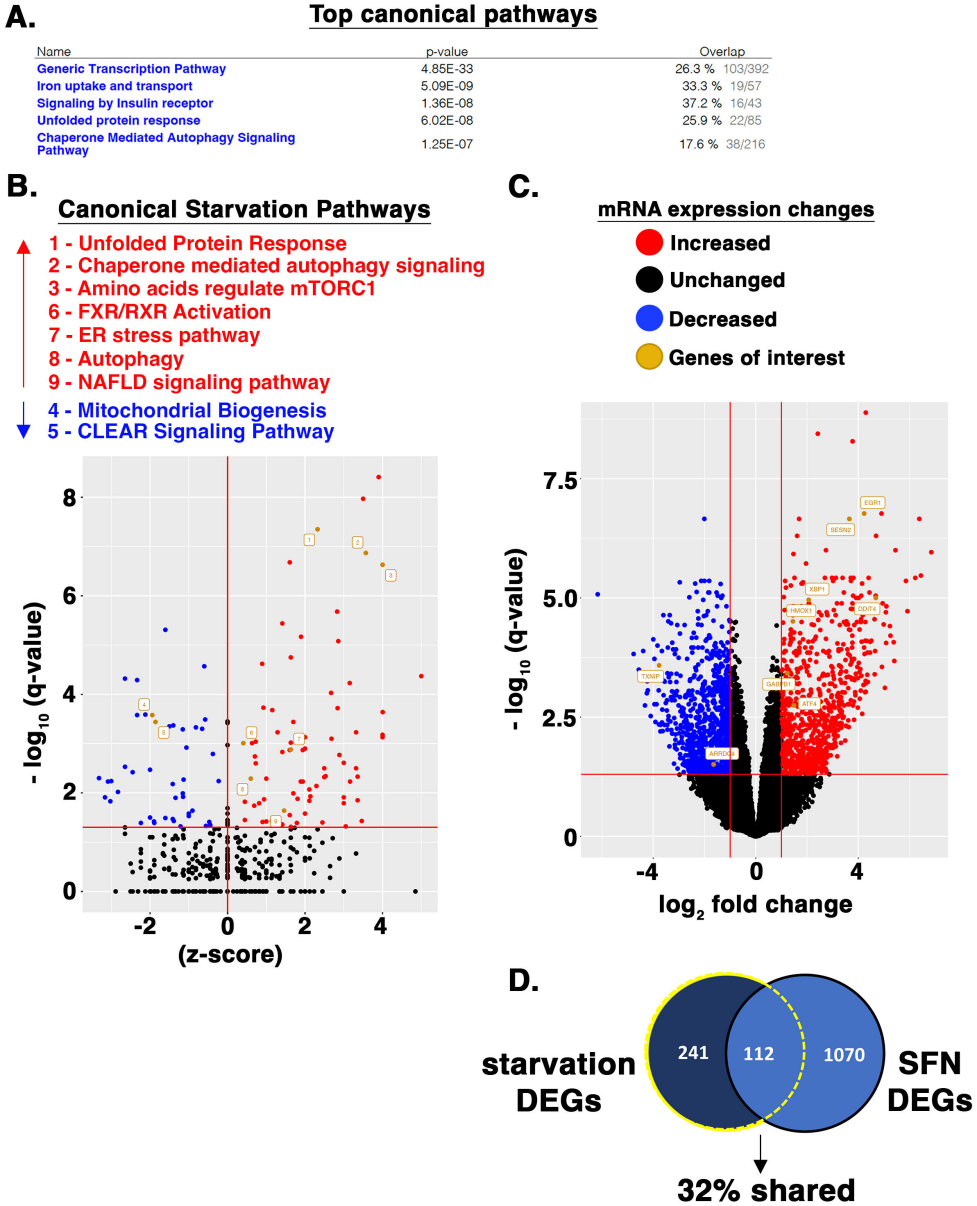
SFN induces starvation-responsive transcriptional programs. **(A)** RPE-1 cells were treated with DMSO or SFN for 4 h after which RNA was isolated and subjected to RNA-seq analysis. Listed are the top canonical pathways impacted according to IPA along with *p*-values and the fraction of pathway genes changed for each pathway. **(B)** Volcano plot showing elevated (red dots and text) and suppressed (blue dots and text) canonical pathways associated with starvation as identified by IPA. **(C)** Volcano plot highlighting expression changes in specific genes that were analyzed in various other assays or discussed throughout the manuscript. Red dots represent increased mRNA expression, blue dots represent decreased mRNA expression, and black dots represent unchanged. Orange dots and name tags denote genes of interest. **(D)** SFN and HeLa cell starvation RNA-seq datasets were filtered to DEGs as defined by having an adjusted p-value (i.e., q-value) < 0.05 and a log2FC < −1 or log2FC > 1. 353 DEGs (242 + 112) from the starved HeLa cells and 1,182 DEGs (1,070 + 112) from the SFN treated RPE-1 cells met these criteria. The DEG list from both analyses were compared via Venn diagram and the number and percentage of DEGs shared between the two data sets is displayed.

We further interrogated the transcriptional starvation responses elicited using RNA-seq analysis of RPE-1 cells treated with SFN for 4 h (raw data file accessible via GEO number GSE275316). Ingenuity Pathway Analysis (IPA) showed that the top canonical pathways impacted were general transcription machinery, iron uptake and transport, signaling by insulin receptor, unfolded protein response, and chaperone-mediated autophagy signaling (Figure 7A). Gene ontology (GO) enrichment analysis of canonical starvation pathways showed that SFN reduced mRNA expression of many components of general transcription, but selectively activated a number of transcriptionally-modulated stress and starvation-related response pathways including the UPR, ER stress, autophagy, and amino acid regulation of mTORC1 (Figure 7B). Highly induced genes included chaperones (i.e., heat shock proteins A1A, A1B, and A6), numerous ATG genes of the autophagy pathway as well as *XBP1* (see Supplementary Table S3 – list of differentially expressed genes (DEGs) ranked by *p*-value). In addition to playing a central role for the UPR, XBP1 is a nexus for directing responses to starvation (60).

Four hours of SFN treatment decreased mitochondrial biogenesis pathway as identified by IPA (Figure 7B), although there was robust induction of GA binding protein transcription factor (*GABPB1*) (Figure 7C), an inducer of gene targets that promote mitochondrial biogenesis (61). The CLEAR signaling pathway was also suppressed at 4 h, based on decreased mRNA expression of the CLEAR inducing transcription factors, TFEB and MITF. Yet, 24 h of SFN increased *TFEB* mRNA expression along with a set of CLEAR genes (Figure 3E). Notably, Cesana et al. observed that it took 6 h for *TFEB* to be transcriptionally upregulated in starved HeLa cells and that the early growth response 1 (EGR1) protein promotes TFEB transcriptional activity (62). *EGR1* mRNA was robustly increased (> 16-fold) by 4 h of SFN (Figure 7C). Additional changes consistent with a nutrient deprivation response were upregulation of several inhibitors of mTOR including DNA damage-inducible transcript 4 protein (*DDIT4* (aka REDD1); >16-fold increase) and *sestrin 2* (> 8-fold increase) (Figure 7C), confirming the reduced mTOR signaling and elevated sestrin 2 detected by Western blotting and qPCR (Figure 2). Both of these mTORC1 inhibitors are downstream targets of the ATF4 transcription factor (63), which was likewise elevated (Figures 6D, 7C). These RNA-seq data show that acute SFN treatment induces numerous transcriptional responses elicited by fasting.

## Discussion

Across species, caloric restriction (CR) without malnourishment extends lifespan and healthspan by activating pathways that collectively improve organismal resilience at the cellular level. These include autophagy, DNA repair and maintenance, mitochondrial and lysosomal biogenesis, and stress neutralization (e.g., glutathione synthesis, xenobiotic detoxification) (64). Numerous approaches to achieving CR have been tested, albeit with mixed results. These approaches work well in laboratory environments in which experimentalists control animal adherence to dietary restrictions and eating patterns/timing, but compliance among humans is more challenging and represents a major confounding factor in clinical trials [e.g., (65, 66)].

**Figure 8:**
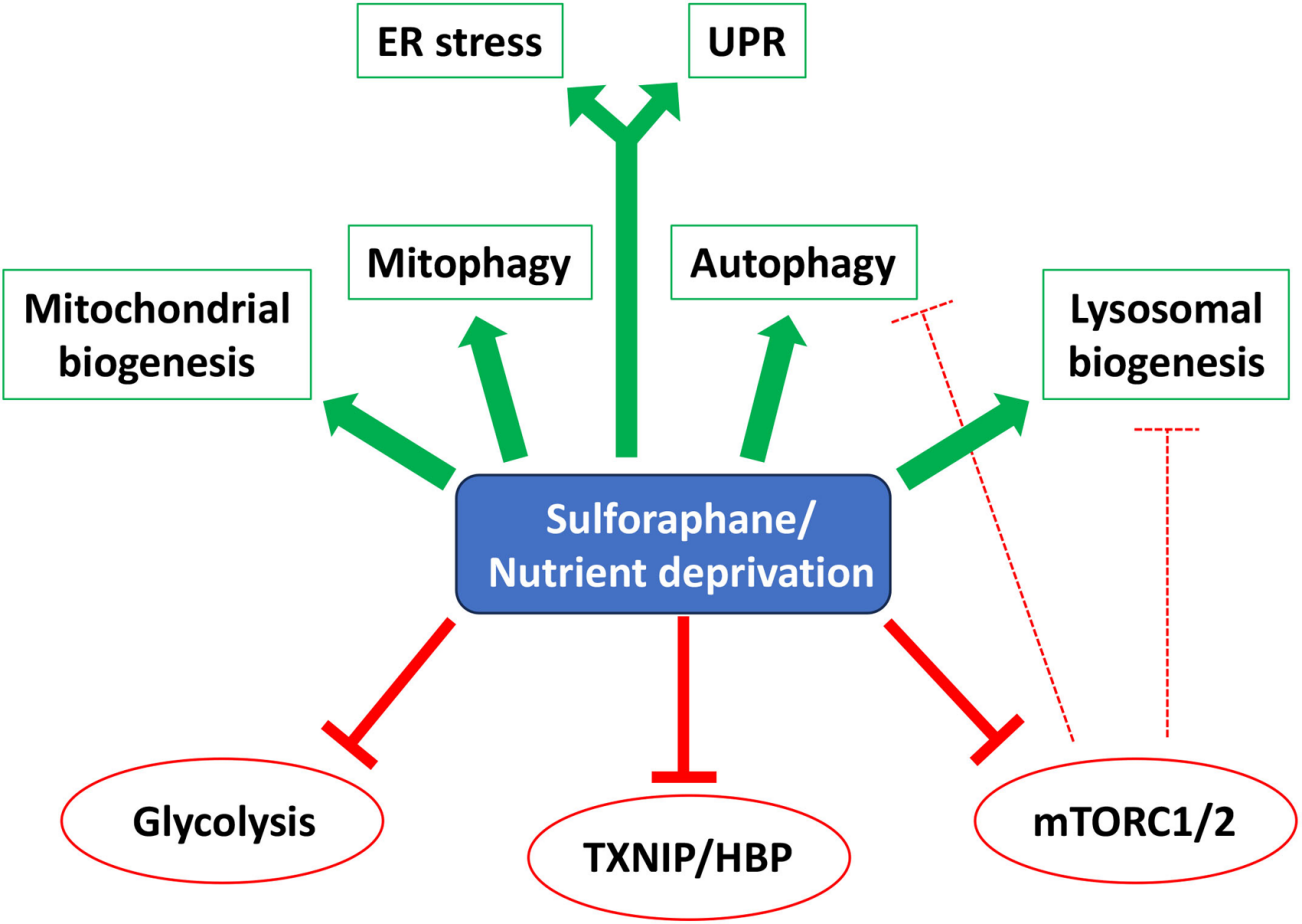
SFN is a starvation/CR mimetic. Model showing overlapping pathways affected by SFN and starvation/Caloric Restriction (CR). Green arrows indicate activation and red ‘T’ symbols (solid and hashed) indicate inhibition. Abbreviations: Endoplasmic Reticulum (ER) Stress, Unfolded Protein Response (UPR), Hexosamine Biosynthetic Pathway (HBP).

Alternative strategies to CR include fasting mimetics that elicit therapeutic benefits without necessarily reducing caloric or macronutrient consumption. Natural compounds and derivatives characterized as CR mimetics include rapamycin (mTOR inhibitor), metformin (AMPK activator), 2-DG (glycolysis inhibitor), resveratrol and nicotinamide (activators of sirtuins), spermidine (regulator of proteome acetylation), curcumin (anti-oxidant), and fungal polysaccharides, among others [reviewed in (64)]. Our data show that SFN treatment acutely elicits hallmarks of a CR mimetic (see Figure 8 model), mirroring aspects of AA, glucose, and growth factors deprivation. Because SFN modifies susceptible cysteine residues on multiple proteins, the effects of the phytochemical represent the sum of the pathways directly modified plus any accompanying downstream cellular adaptations. As such, SFN activates *select* responses rather than the full repertoire of cellular starvation responses. We observed that SFN: (1) preserves mitochondrial membrane potential, limits mitochondrial matrix oxidation, promotes homeostatic mitophagy, and adaptively induces mitochondrial biogenesis to increase mitochondrial mass; (2) acutely suppresses insulin and mTORC2 signaling and adaptively decreases mTORC1 signaling, (3) amplifies autophagic flux and cargo clearance, (4) activates programs to increase lysosome abundance, (5) adaptively increases glucose uptake and a proxy of PDH activity to increase acetyl-CoA entering the TCA cycle, and (6) activates signature starvation-responsive transcriptional programs. Notably absent was a sustained activation of AMPK (data not shown), a mechanism invoked during an energy crisis arising from a deficiency of ATP (67, 68).

We identified novel mechanisms by which SFN functions as a CR mimetic in the context of TXNIP-regulated glucose uptake. TXNIP increases GLUT endocytosis from the plasma membrane to restrict extracellular glucose uptake (49, 51). In the absence of insulin signaling, such as during starvation or exercise, TXNIP protein levels increase (69, 70). Yet, if these scenarios lead to ATP shortages, AMPK phosphorylates TXNIP to mark it for degradation allowing increased cellular glucose uptake to restore bioenergetic homeostasis (49). At the transcriptional level, *TXNIP* mRNA expression is induced when levels of the glycolytic intermediates G6P (52) or glyceraldehyde-3-phosphate (71) or adenine nucleotides (72) accumulate above a threshold. Glucosamine-6-phosphate produced by the HBP can also induce *TXNIP* gene expression (58).

In RPE-1 cells, SFN initially decreased glucose uptake (Figure 4A), likely due to the rapid but acute disruption of Akt signaling (Akt pThr308, pSer473) (Figures 2B,C), followed by a compensatory decrease in TXNIP to restore glucose uptake (Figures 5A,C, respectively). This reduction of *TXNIP* at the mRNA level was partially attributable to blocking the MondoA/Mlx heterodimer from occupying the *TXNIP* promoter (Figure 5D), although this was not a consequence of SFN preventing MondoA and Mlx heterodimer formation (Supplementary Figure S1C) or altering levels of MondoA or Mlx protein amounts (Figure 5A). Notably, in a mouse photoreceptor-derived cell culture line challenged with advanced glycation end products that elevated *TXNIP* mRNA, SFN suppressed this induction through the AMPK pathway (73). We did not observe AMPK activation but did find that supplementing cells with exogenous glucosamine reversed *TXNIP* mRNA suppression by SFN (Figure 6A), consistent with the isothiocyanate inhibiting GFAT of the HBP. Additional evidence supporting this interpretation is that pharmacological inhibition of GFAT reduced *TXNIP* mRNA similarly to SFN (Figure 6B) and this reduction was likewise reversed by glucosamine supplementation (Figure 6C). Further evidence consistent with GFAT inhibition by the phytochemical stems from SFN elevating levels of F6P (Figure 4C), the substrate for GFAT.

SFN also inhibited insulin-PI3K-PDK1-Akt signaling in RPE-1 cells within minutes (Figure 2), but this inhibition resolved and Akt signaling recovered by 8 h and was sustained at 24 h, as indicated by phosphorylation of PRAS (pPRAS), a substrate and functional readout of active Akt (Supplementary Figure S1E). This restored Akt activity was coincident with a dramatic suppression of TXNIP protein expression at 8 and 24 h (Figure 5A and (51)) leading to increased glucose uptake as an adaptive response at 24 h (Figure 4A). Thus, SFN initially reduces glucose uptake but this transient effect resolves, and the cellular uptake of glucose recovers over time coincident with the suppressed expression of *de novo* TXNIP. Notably, circulating free fatty acids such as those resulting from consuming a Western diet or having diabetes, induce mitochondrial ROS in muscle, which leads to over-expression of TXNIP, reduced glucose uptake, and hyperglycemia (74). These findings along with our work indicate that the anti-hyperglycemic efficacy of SFN (75) likely stems from a combination of suppressing ROS (via Nrf2 activation) and suppressing TXNIP (Nrf2-independent). Consistent with our RPE-1 cell culture data, human studies have shown anti-hyperglycemic effects and reduced HbA1c elicited by SFN in obese patients with poorly-managed type 2 diabetes [e.g., (19)]. Increased turnover of intracellular components by autophagy is a hallmark of CR and CR mimetics [e.g., (76)] and SFN promoted autophagic flux (Figures 3B–D) accompanied by the induction of CLEAR genes (Figure 3F), increased lysosomal mass (Figures 3A,E), and increased mitophagy (Figure 1D). These data support a model in which SFN inhibits mTORC1 (Figure 2D), leading to activation of the EGR1 and TFEB transcription factors to induce autophagy and the CLEAR transcriptional program for lysosome biogenesis (Figure 3), extending previous studies of SFN-promoted autophagic flux (40) and induction of the CLEAR network in HeLa, Hep2G, and 1,321 N1 human astrocytoma cells (43).

Akin to the challenges currently facing the therapeutic applications of caloric restriction and fasting (13), identifying the dose, dosing frequency, and route of administration for SFN in each particular disease setting and patient population constitutes a primary challenge in demonstrating consistent and reliable efficacy (26). Resolving these dosing questions may be facilitated by our finding that SFN augmented autophagy beyond the robust induction achieved by rapamycin and nutrient deprivation (Figure 3C). The maximal responses elicited by SFN support the consideration of employing pulsatile dosing regimens to remove and replace damaged macromolecules and organelles while retaining cellular health and survival through periods of SFN holidays (77). In alignment with this notion, we previously showed that administering SFN three times a week (Monday, Wednesday, and Friday) for 3 months recovered cone function in mice undergoing RPE oxidative stress (78).

A comparison of RNA-seq from HeLa cells starved of all nutrients for 4 h by Galves et al. (79) versus our RNA-seq data from 4 h of SFN treatment revealed that 32% of the DEGs changed 2-fold or greater by starvation were likewise changed by SFN (Figure 7D). Notably, SFN and complete starvation both induced responses related to ER stress, the UPR, autophagy, as well as inhibition of mTOR signaling. Galves et al. highlighted a list of 17 activated genes they termed known ‘starvation-genes’ and SFN induced 10 of these (*ATF3*, *ZFP36*, *EIF2AK3*, *ZFYVE1*, *ASNS*, *DDIT3*, *GABARAPL1*, *NFE2L2*, *XBP1*, and *ATF4*), decreased none, and did not statistically significantly alter the expression of 7 (*RRAGC*, *MTMR3*, *FNIP1*, ATG14, SIRT1, ULK1, and *KLF10*) (Supplementary Table S3). Because this comparison uses two distinct human epithelial cell lines, the robust overlap between starvation-associated DEGs in the two data sets highlights the conservation of responses to starvation between cell types within a species, consistent with the evolutionary conservation of nutrient deprivation responses across species [e.g., (80)].

The cultured RPE-1 cells used for these studies enabled mechanistic pathway analyses to be done, which were further facilitated by leveraging RPE-1 reporter cell lines for mitochondrial redox status, mitophagy, and autophagy (20, 25). The cultured cells additionally allowed for the application of pharmacological inhibitors to rapidly inhibit insulin signaling and HBP, circumventing *in vivo* challenges including metabolism by the liver (i.e., first pass effect) and determining the most efficient routes of administration to reach target cells or tissues. Caveats of using cultured cells in these studies include not addressing tissue context, age, sex, health, or morbidities. An additional consideration is that the effects of SFN *in vivo* could vary depending on the specific cell type being studied (e.g., adipocytes versus RPE) and the local metabolic milieu. Consistent with our autophagy and mitophagy findings, SFN increased the autophagy of lipids (aka, lipophagy) in 3 T3-L1 adipocytes by inhibiting mTOR (81) and induced adipocyte browning and glucose uptake (82). The phytochemical also inhibited adipogenesis and reduced obesity through AMPK pathway activation *in vivo* in mice fed a high-fat, obesogenic diet (11). Importantly, the reported physiological outcomes of SFN *in vivo* are largely consistent with the cellular impacts of the phytochemical that we report here.

Although we did not definitively identifying any new substrates directly modified by the phytochemical, the findings of this study advance our understanding of SFN by implicating potential new candidate targets including PDK1 (Figure 2C), phosphofructose kinase (Figure 4C), PDH kinase (Figure 4E), MondoA/Mlx (Figure 5D), and GFAT (Figure 6A). Moreover, the recognition of SFN as a fasting mimetic provides a plausible mechanistic explanation for many of the physiological effects of the compound (e.g., reduced hyperglycemia and resistance to mitochondrial dysfunction) and could be useful for informing dosing strategies to circumvent starvation-associated toxicities.

## Data Availability

The datasets presented in this study can be found in online repositories. The names of the repository/repositories and accession number(s) can be found in the article/Supplementary material.

## Supplementary material

Supplementary Figure S1 (A) RPE-1 cells were starved of glucose for the indicated times. Representative graph of the fold change response of TXNIP mRNA levels (detected by qPCR). RPE-1 cells were incubated for 8 h in the indicated concentrations of glucose. Protein lysates were analyzed by anti-TXNIP Western blotting. (B) RPE-1 cells were treated with DMSO (blue trace) or SFN (red trace) as well as with 1 μM Actinomycin D to block de novo TXNIP mRNA synthesis for the time indicated. TXNIP mRNA levels were analyzed by qPCR. (C) MondoA Western blots of RPE-1 cell lysates (Inputs) and co-immunoprecipitation (IPs) with an antibody to Mlx. Cells were treated for 0, 4, or 8 h with SFN. MondoA expression is shown in both a short and longer exposure to facilitate visualization of the Inputs. (D) RPE-1 cells were treated with siCON or siNrf2 for 2 days and then harvested to quantify Nrf2 mRNA levels by qPCR. (E) Samples from Figure 5A were probed for pPRAS and actin. ****indicates statistical significance (*p* = 0.0002). The migration of molecular weight markers (kDa) is shown to the left of blots (A, C, and E).

Supplementary Table S1 List of chemicals, reagents, and antibodies, companies, catalog numbers, and concentrations/dilutions used in these studies.

Supplementary Table S2 List of qPCR primers used in these studies.

Supplementary Table S3 List of DEGs (ranked by *p*- value) with a p-value ≤ 0.05 derived from RNA-seq of RPE-1 cells treated with either DMSO (vehicle) or SFN for 4 h (*n*= 5 samples/treatment). Yellow highlighting of gene symbols (Column H) marks individual DEGs discussed in the text.
