# Supplementary figures and images for: Sulforaphane acutely activates multiple starvation response pathways
Source: Front Nutr. 2025 Jan 6;11:1485466. doi: 10.3389/fnut.2024.1485466 (PMC11758633; doi:10.3389/fnut.2024.1485466)

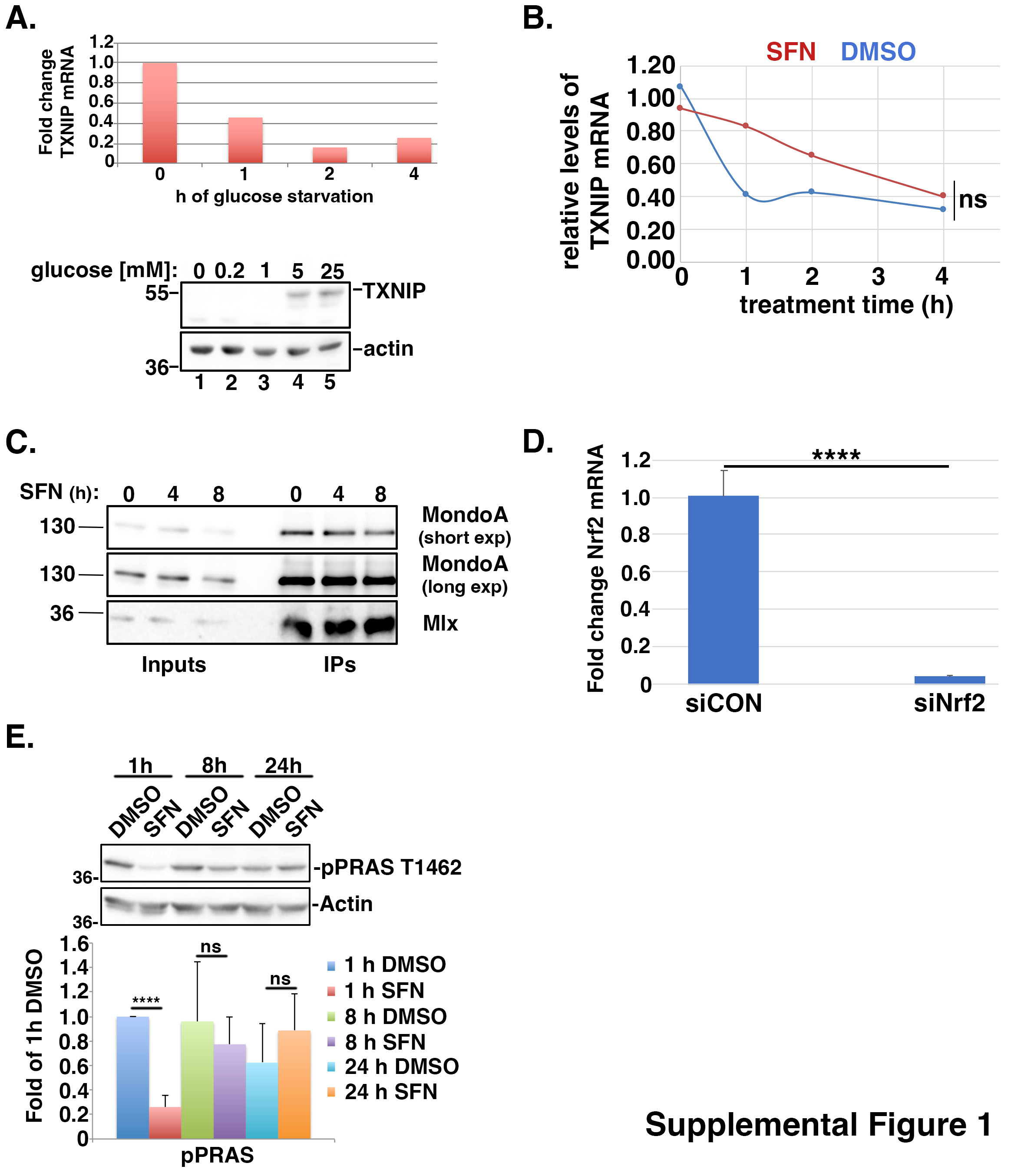

Supplement: Supplementary Figure S1 — (A) RPE-1 cells were starved of glucose for the indicated times. Representative graph of the fold change response of TXNIP mRNA levels (detected by qPCR). RPE-1 cells were incubated for 8 h in the indicated concentrations of glucose. Protein lysates were analyzed by anti-TXNIP Western blotting. (B) RPE-1 cells were treated with DMSO (blue trace) or SFN (red trace) as well as with 1 μM Actinomycin D to block de novo TXNIP mRNA synthesis for the time indicated. TXNIP mRNA levels were analyzed by qPCR. (C) MondoA Western blots of RPE-1 cell lysates (Inputs) and co-immunoprecipitation (IPs) with an antibody to Mlx. Cells were treated for 0, 4, or 8 h with SFN. MondoA expression is shown in both a short and longer exposure to facilitate visualization of the Inputs. (D) RPE-1 cells were treated with siCON or siNrf2 for 2 days and then harvested to quantify Nrf2 mRNA levels by qPCR. (E) Samples from Figure 5A were probed for pPRAS and actin. ****indicates statistical significance (p = 0.0002). The migration of molecular weight markers (kDa) is shown to the left of blots (A, C, and E). [file Image_1.jpeg]
